# Vitreous chamber depth as part of axial elongation in pediatric myopia

**DOI:** 10.1186/s12886-026-04622-1

**Published:** 2026-01-16

**Authors:** Sinan Liu, Ming Li, Yihua Zhu, Huihang Wang

**Affiliations:** 1 Department of Ophthalmology, The First Affiliated Hospital, Fujian Medical University, No.20 Cha Zhong Road, Fuzhou, 350005 China; 2 Department of Ophthalmology, National Regional Medical Center, Binhai Campus of The First Affiliated Hospital, Fujian Medical University, Fuzhou, China; 3 Fujian Institute of Ophthalmology, The First Affiliated Hospital, Fujian Medical University, Fuzhou, China; 4 Fujian Provincial Clinical Medical Research Center of Eye Diseases and Optometry, The First Affiliated Hospital, Fujian Medical University, Fuzhou, China; 5 School of Smart Marine Science and Engineering, Fujian University of Technology, Fuzhou, China; 6 Fujian Provincial Key Laboratory of Marine Smart Equipment, Fuzhou, China

**Keywords:** Myopia progression, Axial length, Vitreous chamber depth, Pediatric ophthalmology

## Abstract

**Background:**

This study explored the connections between axial length (AL) and other ocular parameters, including central corneal thickness (CCT), aqueous chamber depth (AD), lens thickness (LT), and vitreous chamber depth (VCD), during myopia progression in children.

**Methods:**

This study was conducted on a Chinese pediatric population, comprising 42 participants in a prospective cohort and 406 participants in a retrospective study. Standardized protocols were used to measure ocular parameters. Statistical analyses included descriptive statistics, t-tests, ANOVA, and correlation analyses to compare parameters across AL subgroups.

**Results:**

Significant positive proportional relationships were observed between AL and VCD (R² = 0.97), and moderate correlations with AD (R² = 0.49) and LT (R² = 0.30). No significant correlation was found between AL and CCT (R² = 0.00). Longer AL was associated with increased VCD and AD and a decrease in LT. Regular monitoring of AL, and particularly VCD, provides a sensitive measure of ongoing myopia progression and can be used to track its dynamics.

**Conclusions:**

VCD is the primary component associated with AL elongation in pediatric myopia, with minimal contributions from AD and LT. Regular monitoring of AL and VCD may improve the assessment of myopia progression and support personalized intervention strategies.

## Introduction

Myopia has emerged as a significant public health issue worldwide, with particularly high prevalence rates in Asian countries [1]. According to the latest literature, high myopia is especially prevalent in the developed regions of East and Southeast Asia [2, 3]. Studies have also shown that incident myopia is associated with childhood myopia, East Asian ethnicity, female sex, less time spent outdoors, and parental myopia [4, 5]. In East Asia, childhood myopia is more prevalent and requires continuous management to address potential risks to children’s vision health [6, 7]. The increasing incidence of childhood myopia is a pressing concern, as it often progresses into high myopia, elevating the lifetime risk of severe ocular complications such as retinal detachment, glaucoma, cataract, and myopic maculopathy [8]. The consequences of myopia, particularly high myopia, are especially profound among younger populations, as it reduces productive working years, increases the costs of corrective measures, and imposes a significant economic burden on society [7, 9].

Axial length (AL) is an important indicator of myopia progression [10–12]. The elongation of AL is closely related to changes in several ocular components, including aqueous chamber depth (AD), lens thickness (LT), and vitreous chamber depth (VCD). In contrast, central corneal thickness (CCT) remains relatively stable during axial elongation, with minimal significant changes despite axial growth. These structural changes interact during the process of axial elongation and collectively affect the eye’s refractive state [13]. Existing studies have shown that corneal thickness remains relatively stable during axial elongation, with minimal significant changes despite axial growth [14]. In contrast, AD gradually increases with axial elongation, especially during childhood and adolescence [15]. Changes in AD are essential for the eye’s optical function and refractive state. Lens thickness follows a dynamic pattern: it gradually thins during childhood but increases with continued axial elongation, serving as a compensatory mechanism to help maintain the eye’s refractive power. VCD is closely associated with AL elongation and constitutes its largest anatomical component [16]. Studies have shown that patients, especially those with high myopia, have an axial elongation throughout their lives [17, 18]. During the active growth period in childhood and adolescence, the observed axial elongation is primarily due to an increase in VCD.

Despite considerable research on the relationship between these ocular components and axial elongation [14, 19], several gaps remain in the current literature. Specifically, there is a lack of longitudinal studies examining the interactions among these ocular components, particularly across different age groups and refractive status categories. Most studies focus on individual ocular components and primarily involve adult or adolescent populations, with limited investigation into the axial growth process in early childhood and adolescence [20]. The present study addresses these gaps through a unique dual-design approach, combining an extensive, stratified retrospective analysis with a longitudinal prospective cohort. This methodology enables a comprehensive examination of the dynamic interactions between ocular components across a wide pediatric age range (3–18 years) and the full spectrum of axial lengths, providing novel insights into the anatomical process of axial elongation during childhood.

## Methods

### Study participants and eligibility criteria

This study employed a dual-approach design, comprising a retrospective analysis and a prospective cohort study. A large retrospective sample (*n* = 406 eyes) was stratified into AL groups (20–21 mm, 22–23 mm, 24–25 mm, 26–27 mm, > 27 mm) to analyze differences in ocular parameters (CCT, AD, LT, VCD) across AL ranges, cross-sectional data only (single time-point measurements). A prospective cohort study involving 42 pediatric patients aged 3 to 18 years without ocular disease, with complete biometric data collected in 2022, included longitudinal measurements with 1-year follow-up intervals. Participants in both studies were required to have complete biometric data records for both eyes. Moreover, we excluded individuals with any of the following: a history of ocular surgery (including refractive surgery and cataract surgery), significant ocular pathology or media opacities (e.g., cataract, corneal scar, glaucoma, or retinal disease) that could impair biometric measurement, and systemic diseases known to affect ocular growth or refraction (e.g., diabetes, Marfan syndrome, or Down syndrome).

### Data collection

Data were gathered with a detailed ophthalmic examination, including the measurement of multiple ocular biometric parameters. The spherical equivalent (SE) was sphere + ½ cylinder. Refractive error was measured under full cycloplegia prior to examination, using an automatic refractometer (TOPCON, Japan), ensuring complete relaxation of accommodation and the measurement of the objective refractive status. AL, CCT, AD, LT, and VCD were measured using the IOLMaster 700 (Carl Zeiss Meditec, Jena, Germany). All measurements were performed by the same experienced technician under consistent conditions to ensure data consistency and comparability.

### Statistical analysis

Statistical analysis was performed using SPSS (Version 26) to compare the differences in ocular biometric parameters between the two studies and across different AL ranges. Data normality was assessed using Shapiro-Wilk tests. Descriptive statistics are presented as mean ± standard deviation for normally distributed data. To compare ocular biometric parameters between the two study designs and across different AL ranges, independent samples t-tests were used. For multiple comparisons, one-way analysis of variance (ANOVA) was used for normally distributed data, and the Bonferroni correction was applied. For non-normally distributed parameters, the Mann-Whitney U test and Kruskal-Wallis H test were applied as non-parametric alternatives. Correlation coefficients (R²) and 95% CI are also given. A two-tailed *P-*value of < 0.05 was considered statistically significant for all analyses.

## Results

### Comparative analysis of study designs

In this study, we used comparative analysis to examine data from a cohort study (*n* = 42) and a retrospective study (*n* = 406) in Table 1. The results indicated that there were significant differences in demographic characteristics and ocular parameters between the two groups. The cohort study included younger children (mean age 5.9 ± 1.6 years) with a narrower age range, while the retrospective study had a wider age range (3–18 years, mean 8.0 ± 3.3 years). Female participants comprised 47.6% of the cohort study and 50.2% of the retrospective study. Ocular parameters also differed: SE was − 0.51 ± 1.47 D in the cohort study versus − 0.93 ± 1.81 D in the retrospective study, indicating that the retrospective study group, on average, had more negative refractive error. AL was 23.04 ± 1.43 mm in the cohort study and 23.51 ± 1.35 mm in the retrospective study. Other parameters, including CCT, AD, LT, VCD, and the ratio of VCD to AL (VCD/AL), showed minor differences between the groups.

**Table 1:** Demographic and ocular characteristics

Characteristic	Cohort Study (*n* = 42)	Retrospective Study (*n* = 406)
Females, %	47.6	50.2
Age (years)	5.90 ± 1.60	8.00 ± 3.30
3–5, n, %	18 (42.9)	99 (24.4)
6–8, n, %	21(50.0)	153 (37.7)
9–11, n, %	3 (7.1)	92 (22.7)
12–14, n, %	0 (0.0)	37 (9.1)
15–18, n, %	0 (0.0)	25 (6.2)
SE (D)	−0.51 ± 1.47	−0.93 ± 1.81
AL (mm)	23.04 ± 1.43	23.51 ± 1.35
20–21, n, %	3 (7.1)	5 (1.2)
21–22, n, %	9 (21.4)	49 (12.1)
22–23, n, %	10 (23.8)	104 (25.6)
23–24, n, %	15 (35.7)	115 (28.3)
24–25, n, %	3 (7.1)	73 (18.0)
25–26, n, %	0 (0.0)	44 (10.8)
26–27, n, %	2 (4.8)	11 (2.7)
> 27, n, %	0 (0.0)	5 (1.2)
CCT (µm)	540.02 ± 28.71	543.93 ± 33.03
AD (mm)	2.93 ± 0.28	3.05 ± 0.32
LT (mm)	3.63 ± 0.24	3.56 ± 0.25
VCD (mm)	15.88 ± 1.23	16.36 ± 1.28
VCD/AL	0.69 ± 0.02	0.70 ± 0.02

Data are presented as mean ± standard deviation (SD) for continuous variables

*SE* spherical equivalence, *AL* axial length, *CCT* corneal thickness, *AD* aqueous chamber depth, *LT* lens thickness, *VCD* vitreous cavity depth

### Cross-sectional analysis of ocular biometrics by AL group

Our cross-sectional analysis of the retrospective cohort (*n* = 406), stratified by AL range, 20–21 mm (*n* = 5), 21–22 mm (*n* = 49), 22–23 mm (*n* = 104), 23–24 mm (*n* = 115), 24–25 mm (*n* = 73), 25–26 mm (*n* = 44), 26–27 mm (*n* = 11), and > 27 mm (*n* = 5), revealed distinct patterns in the relationship between AL and other ocular structural parameters (Table 2). The differences in means between successive AL groups (denoted as Δ) were calculated to model the component contributions to axial elongation. We found that the comparison between the shortest (20–21 mm) and longest (27 mm) AL groups showed statistically significant differences in AD, VCD, and LT (*P* < 0.0001, *P* < 0.0001, and *P* = 0.0312, respectively). As AL increased from 20 to 21 mm to > 27 mm, the mean AD increased from 2.52 mm to 3.45 mm, the mean VCD significantly increased from 13.94 mm to 20.28 mm, while the mean LT decreased from 3.84 mm to 3.44 mm. Moreover, the relationship between AL increment (ΔAL) and increments in AD, LT, and VCD (ΔAD, ΔLT, ΔVCD) shows that the increase in VCD accounts for a large proportion of the growth in AL, with smaller contributions from AD and LT. The proportion of VCD change to AL change (X) was 85.5% to 98.3%, respectively, indicating that VCD was the primary factor contributing to axial elongation. The joint effect of AD and LT in relation to AL variation (Y) was, in general, even lower, between 1.6% and 15.1%, indicating the small contribution of the two parameters. The individual contribution of AD to AL change (Z) was also small, ranging from − 3.4% to 28.1%.

**Table 2:** Ocular biometric parameters across different axial length ranges

AL Range (mm)	AL (mm) Mean (95% CI)	AD (mm) Mean (95% CI)	LT (mm) Mean (95% CI)	VCD (mm) Mean (95% CI)	△AL* (mm)	△AD (mm)	△LT (mm)	△VCD (mm)	X^§^	Y^†^	Z^‡^	Y/Z
20–21	20.82 (20.64 to 21.00)	2.52 (2.39 to 2.66)	3.84 (3.57 to 4.12)	13.94 (13.66 to 14.21)	-	-	-	-	-	-	-	-
21–22	21.62 (21.54 to 21.70)	2.64 (2.56 to 2.72)	3.81 (3.73 to 3.89)	14.62 (14.53 to 14.72)								
20–21 vs. 21–22					0.79	0.11	−0.034	0.69	86.7	9.8	14.2	69.4
22–23	22.54 (22.48 to 22.59)	2.89 (2.85 to 2.94)	3.69 (3.65 to 3.73)	15.41 (15.35 to 15.47)								
21–22 vs. 22–23					0.92	0.26	−0.12	0.79	85.5	15.1	28.1	53.7
23–24	23.57 (23.52 to 23.62)	3.10 (3.06 to 3.14)	3.50 (3.46 to 3.54)	16.43 (16.36 to 16.49)								
22–23 vs. 23–24					1.03	0.21	−0.19	1.01	97.8	1.6	20.1	7.9
24–25	24.45 (24.39 to 24.52)	3.24 (3.19 to 3.29)	3.41 (3.37 to 3.45)	17.25 (17.16 to 17.34)								
23–24 vs. 24–25					0.88	0.14	−0.092	0.80	90.3	5.3	15.7	33.8
25–26	25.35 (25.28 to 25.43)	3.32 (3.25 to 3.38)	3.39 (3.45 to 3.43)	18.10 (18.02 to 18.18)								
24–25 vs. 25–26					0.90	0.077	−0.021	0.85	94.2	6.2	8.5	73.0
26–27	26.45 (26.29 to 26.61)	3.49 (3.36 to 3.63)	3.36 (3.23 to 3.49)	19.05 (18.88 to 19.22)								
25–26 vs. 26–27					1.09	0.18	−0.036	0.95	86.7	13.0	16.0	81.3
> 27	27.70 (26.92 to 28.47)	3.45 (3.18 to 3.72)	3.44 (3.10 to 3.78)	20.28 (19.23 to 21.32)								
26–27 vs. >27					1.25	−0.042	0.084	1.23	98.3	3.3	−3.4	−99.0
P^**^ *value*	<0.0001	<0.0001	0.0312	<0.0001								

*△AL: Difference in mean AL relative to the preceding axial length range (e.g., AL 21–22 mm vs. 20–21 mm); ^§^X = △VCD /△AL (%), VCD contribution rate, the proportion of VCD change relative to AL change; ^†^Y = (△AD + △LT) /△AL (%); ^‡^Z = △AD /△AL (%); ^**^P values were derived from independent samples t-tests (or Mann-Whitney U tests for non-normally distributed data) comparing the parameter means between the shortest (20–21 mm) and longest (> 27 mm) axial length groups

*AL* axial length, *AD* aqueous chamber depth, *LT* lens thickness, *VCD* vitreous cavity depth

### Annual progression patterns

In this study, we analyzed the annual changes in ocular parameters across different baseline AL ranges using comparative analysis. Cohort data revealed several key trends in Table 3. The yearly changes in AL, AD, LT, VCD, and SE showed variation across different baseline AL ranges. As baseline AL increased from 20 to 21 mm to greater than 24 mm, the annual change in AL initially increased and then slightly decreased, ranging from 0.24 mm to 0.28 mm. The annual changes in AD, LT, and VCD showed fluctuations. Notably, VCD exhibited a wider range of change compared to AD and LT. Furthermore, changes in SE were highly variable, especially when AL > 24 mm; the annual change was − 0.54 D (95% CI: -1.12 to 0.05), indicating more rapid refractive change in eyes with longer AL. Judging by the X, Y, and Z (and Y/Z ratio) results, VCD made a much bigger contribution to the annual changes in AL than AD and LT together.

**Table 3:** Annual changes in ocular biometric parameters across different axial length ranges

Baseline AL (mm)	AL (mm) Mean (95% CI)	AD (mm) Mean (95% CI)	LT (mm) Mean (95% CI)	VCD (mm) Mean (95% CI)	SE (D) Mean (95% CI)	X*	Y^†^	Z^‡^	Y/Z
20–21	0.26 (−0.04to 0.55)	0.06 (−0.10 to 0.22)	−0.09 (−0.29 to 0.11)	0.28 (0.00 to 0.57)	0.11 (−1.76 to 1.98)	111.3	−11.3	23.5	−48.1
21–22	0.28 (0.22 to 0.35)	0.08 (−0.03 to 0.12)	−0.09 (−0.14 to−0.03)	0.30 (0.25 to 0.34)	−0.10 (−0.56 to 0.36)	104.4	−3.1	27.9	−11.0
22–23	0.26 (0.17 to 0.34)	0.09 (−0.06 to 0.11)	−0.09 (−0.14 to−0.05)	0.26 (0.18 to 0.34)	−0.24 (−0.55 to 0.08)	101.1	−1.9	34.3	−5.4
23–24	0.28 (0.19 to 0.37)	0.05 (−0.00 to 0.097)	−0.05 (−0.11 to−0.01)	0.28 (0.19 to 0.37)	−0.37 (−0.63 to−0.11)	100.6	−1.0	17.3	−5.6
>24	0.24 (0.13 to 0.36)	0.05 (−0.00 to 0.11)	−0.04 (−0.12 to 0.04)	0.22 (0.10 to 0.35)	−0.54 (−1.12 to 0.05)	92.2	4.7	21.9	21.4
*P*^§^ value	0.96	0.65	0.61	0.85	0.34	-	-	-	-
All	0.27 (0.23 to 0.31)	0.065 (0.04 to 0.09)	−0.07 (−0.10 to 0.05)	0.27 (0.24 to 0.31)	−0.27 (−0.43 to−0.11)	-	-	-	-

*X = VCD / AL (%); ^†^Y = (AD + LT) / AL (%); ^‡^Z = AD / AL (%); ^§^*P* values were derived from one-way ANOVA (or Kruskal-Wallis test for non-normally distributed data) testing for differences in annual parameter changes across the different baseline AL groups

*AL* axial length, *AD* aqueous chamber depth, *LT* lens thickness, *VCD* vitreous cavity depth, *SE* spherical equivalence

### Structural correlations in myopia

**Fig. 1:**
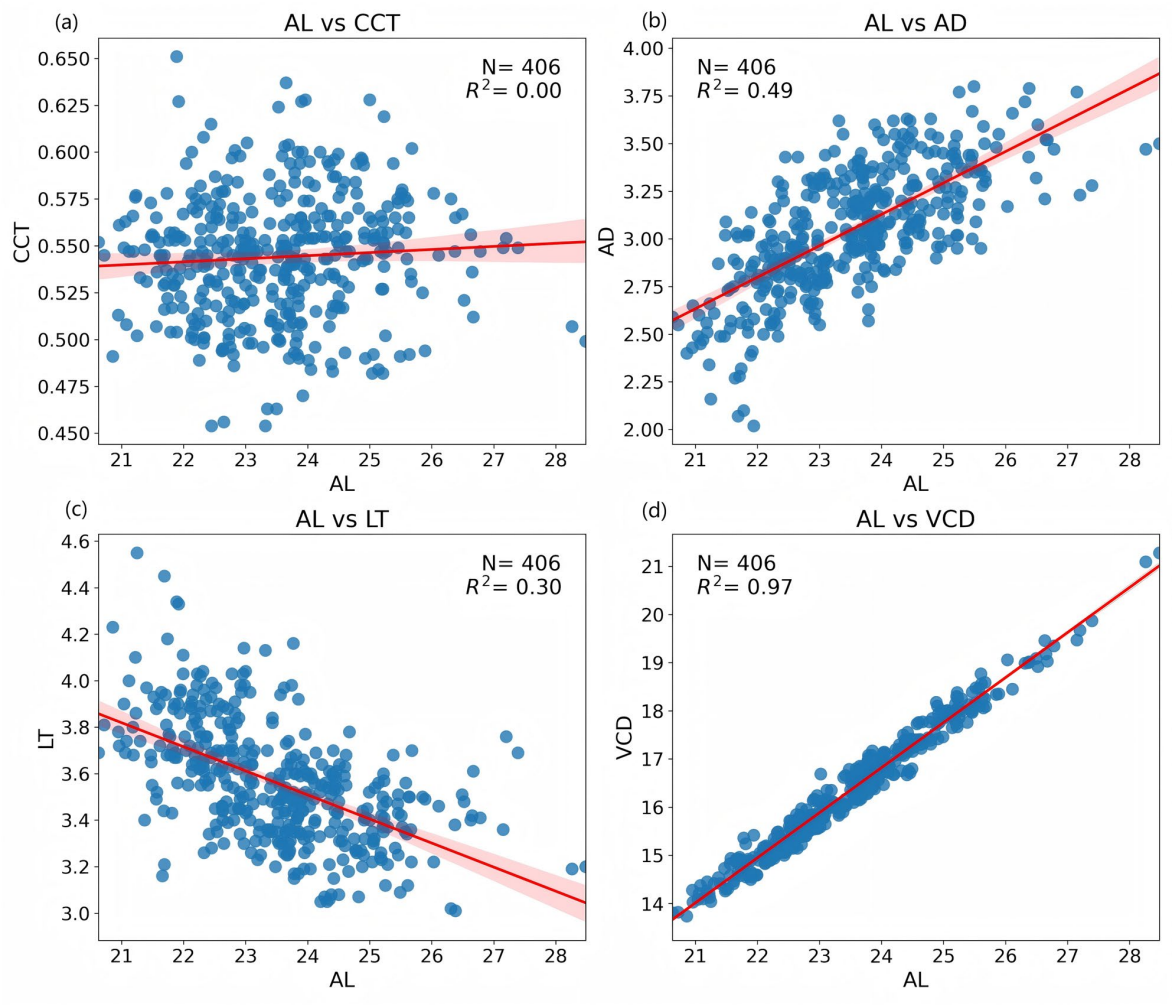
Relationships between (**a**) AL and CCT, (**b**) AL and AD, (**c**) AL and LT, and (**d**) AL and VCD, exhibiting various degrees of variation

We explored the relationships between AL and other ocular parameters through scatter plots with trend lines (Fig. 1), and correlation analysis revealed several key findings. AL showed a strong positive correlation with VCD, as indicated by the high R² value of 0.97, suggesting that VCD increases proportionally with AL. In contrast, AL was moderately correlated negatively with LT (R²=0.30) as LT decreases with increasing AL. The correlation between AL and AD was moderate (R²=0.49), showing a positive relationship where AD increases with AL. Notably, there was no significant correlation between AL and CCT (R²=0.00), implying that CCT remains relatively stable across different AL values.

### Comprehensive analysis of ocular components

**Fig. 2:**
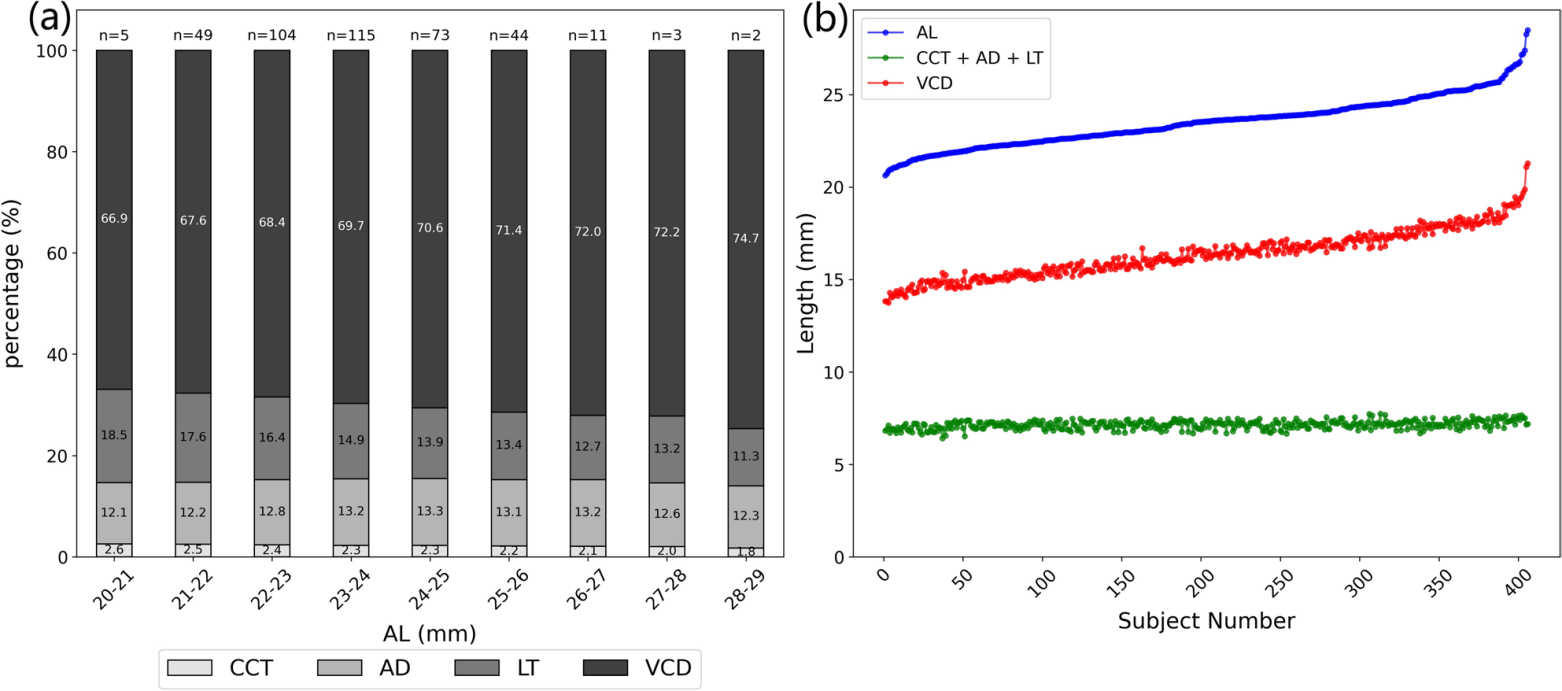
(**a**) Distribution of CCT, AD, LT, and VCD as percentages of total AL across different AL ranges. (**b**) Changes in AL, VCD, and the anterior segment components (CCT + AD + LT) with respect to subject number, along with the relationship between AL and VCD

By analyzing the stacked bar charts and line graphs (Fig. 2), we examined the distribution and trends of ocular parameters across different ranges of AL. The stacked bar chart demonstrated that VCD consistently represented the most significant proportion of AL across all ranges, with its contribution increasing as AL lengthened. In contrast, the combined contribution of CCT, AD, and LT remained relatively stable, accounting for a smaller portion of AL. The trend of the line graph also showed that both AL and VCD had a slight increase with the number of subjects, affirming the predominant contribution of VCD to axial elongation.

### Integrative insights from correlation analysis

**Fig. 3:**
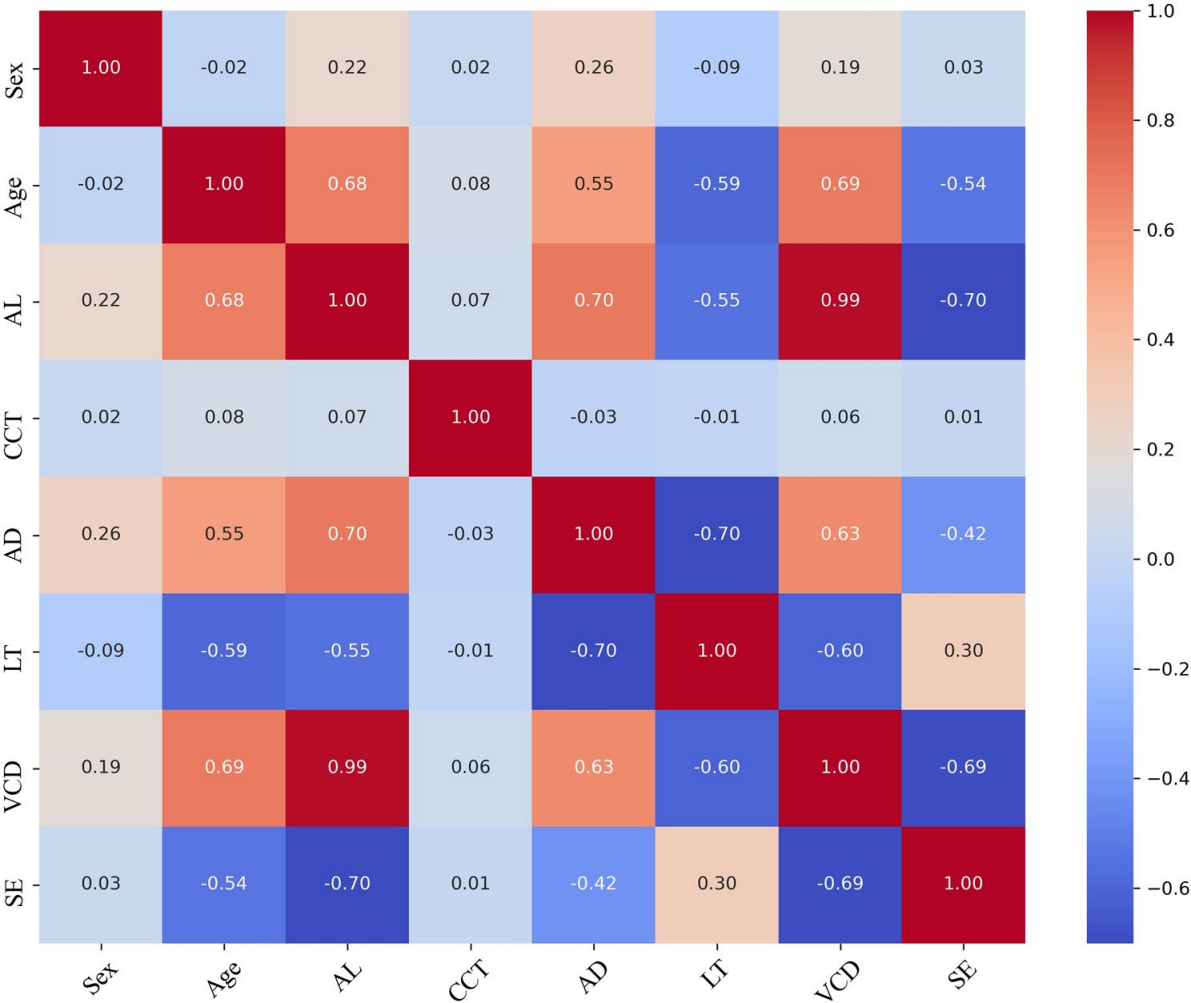
Heatmap depicting the pearson correlation coefficients among biometric parameters

The correlation heatmaps, based on Pearson’s correlation coefficient (r), revealed several key relationships between ocular parameters and demographic factors (Fig. 3). The almost perfect positive correlation between AL and VCD (*r* = 0.99), indicating that these two parameters change almost in tandem; a strong negative correlation between AL and SE (*r* = -0.70) [21], suggesting that increased AL is associated with greater myopia; and moderate to weak correlations between AL and other parameters such as AD (0.70) and LT (*r* = -0.55). Age showed a positive correlation with AL (*r* = 0.68), AD (*r* = 0.55), and VCD (*r* = 0.69), implying that these parameters tend to increase with age. Notably, CCT had weak correlations with other parameters, remaining relatively stable across different conditions.

## Discussion

This dual-design approach reveals a consistent anatomical pattern across both cohorts; VCD showed the strongest association with axial elongation, whereas LT exhibited changes consistent with compensatory modulation. Data from a retrospective study (*n* = 406 eyes) indicated that eyes compensate statically: for longer eyes, the vitreous chamber is proportionately larger and the lens is thinner. The cross-sectional disparity between AL groups was clearly replicated in 42 subjects who were followed over 2 years. This study revealed a significant positive proportional relationship between AL and VCD, with an R² value of 0.97. Additionally, we noted a weak negative correlation between AL and LT, with an R² value of 0.30. There was a moderate positive correlation between AL and AD, with an R² value of 0.49, while no correlation was found between AL and CCT, indicated by an R² value of 0.00. These results suggest that a longer VCD results in longer AL, as is to be expected as the longest anatomical contributor, while LT decreases with longer AL, possibly due to accommodative mechanisms. AD increases with AL but contributes less to its development, and CCT remains stable during axial elongation. However, this relationship is not solely a geometric inevitability but is also influenced by biological mechanisms, such as posterior scleral remodeling. Specifically, the increase in VCD during axial elongation is primarily driven by the expansion and remodeling of the posterior sclera, which increases the vitreous chamber volume. Therefore, the relationship between VCD and AL reflects both geometric factors and biological processes, with geometric factors largely guiding biological relationships. These findings directly reveal the interactions between AL and other ocular parameters during myopia progression and how these interactions vary with age and refractive state.

The positive correlation between AL and VCD likely stems from the vitreous cavity’s expansion during axial elongation [22]. The positive correlation between AL and VCD is mainly attributed to the expansion and remodeling of the posterior sclera, which increases the volume of the vitreous chamber. In cases of pediatric myopia, the vitreous body generally remains attached. However, its direct structural role may decrease after posterior vitreous detachment [23]. On the other hand, the weak negative correlation between AL and LT may relate to the lens’s accommodation mechanis [24]. During myopia development, the lens thins as a compensatory mechanism to partially offset the refractive error induced by axial elongation, but this ability is limited, leading to a gradual decrease in LT with continued AL growth. In addition, the moderate correlation between AL and AD could be due to anatomical relationships in the anterior segment [25]. AD is influenced by factors like corneal curvature and iris morphology, which may be indirectly affected by AL changes. Age-related development of the anterior segment also impacts this correlation. However, there is a lack of correlation between AL and CCT because CCT remains relatively stable during axial elongation, mainly influenced by genetics and local metabolism, independent of the axial growth process.

Compared with previous studies, our findings align with some but differ in others. Research indicates that for children aged 7 to 9, an axial elongation rate of 0.19 mm per year differentiates non-progressive from progressive myopia [26]. Consistent with our findings, across all AL ranges, the AL increases by 0.27 mm per year (95% CI: 0.23 to 0.31), which exceeds the threshold of 0.19 mm per year. Moreover, a strong positive correlation between AL and VCD is consistent with the literature. Similarly, a few studies have shown that children with myopia demonstrated no distinct variations in AD [27]. However, our study stands out with its unique methodological and analytical advances. Whereas previous studies either used a cross-sectional design or examined particular age groups, we conducted a detailed, stratified cross-sectional analysis of a large retrospective cohort (*n* = 406) to map the static relationships among ocular components across AL groups. Separately, a smaller prospective cohort (*n* = 42) was followed longitudinally to track dynamic changes. This approach addresses shortcomings observed in earlier studies, including longitudinal absence of information about interactions between components during active childhood elongation. Crucially, our longitudinal cohort data precisely mirrored the cross-sectional differences between AL groups observed retrospectively, reinforcing the robustness of the static compensation pattern (VCD↑, LT↓) across study designs. The weak negative AL-LT correlation (R²=0.30) also shows variability in the literature [28]; our quantification of LT’s minor negative contribution (ΔLT: -0.034 to -0.021 mm per 1 mm ΔAL) provides clearer anatomical insight than studies reporting no correlation, likely due to our detailed stratification by AL range and longitudinal tracking quantifying rates of change.

Furthermore, our longitudinal correlation between ΔAL and ΔVCD (*r* = 0.99 from cohort data) and our precise quantification of VCD’s proportion (69–98%) of AL change represent advancements over previous estimates. The longitudinal cohort data provide supportive evidence for a correlation between VCD and AL elongation, though the small sample size limits the study’s inferential power. Larger studies are needed to confirm these findings and further explore the relationship over time. By analyzing both cross-sectional distributions and longitudinal progression, we quantified the dynamic contributions of individual ocular components across a broad spectrum of axial lengths in a pediatric population. Specifically, our detailed stratification and longitudinal tracking revealed that LT exhibits a small but consistent negative contribution (ΔLT: -0.034 to -0.021 mm per 1 mm ΔAL). Detailed stratification by AL range may help detect nuanced relationships that can be challenging to capture across studies with different designs or analytical approaches.

Our study reinforces and quantitatively refines the established understanding of ocular biometric changes during pediatric myopia progression. While the correlations between AL, VCD, LT, and AD have been reported in various populations, our findings provide a more granular, dynamic model of their contributions [29, 30]. By precisely quantifying that VCD accounts for 69–98% of the axial elongation and demonstrating a consistent, albeit minor, compensatory thinning of the lens (ΔLT: -0.034 to -0.021 mm per 1 mm ΔAL), we offer a detailed anatomical profile of axial growth.

Consequently, regular monitoring of AL and VCD is valuable for identifying and quantifying the rate of myopia progression, rather than predicting its future onset. This precise tracking can inform management decisions, guiding the timing and intensity of interventions. For children with rapid AL growth, timely preventive measures, such as increased outdoor activity and controlled screen time, are recommended [29, 31, 32]. Understanding AL’s relationships with other parameters can also inform more effective myopia prevention programs, thereby optimizing resource allocation in large-scale screening and intervention efforts to reduce myopia prevalence and associated complications [33, 34].

However, our study has certain limitations. First, the sample was region-specific and focused on children and adolescents aged 3–18, so applicability to other age groups needs verification. Second, the retrospective segment of our data may be subject to selection bias. Finally, while combining cohort and retrospective studies provided rich data, potential information bias and unmeasured confounding factors in the retrospective part cannot be ruled out. Future research directions should involve larger, longer-term longitudinal studies, multicenter and cross-regional collaborations, comprehensive intervention studies, and investigations into the molecular mechanisms underlying AL growth and changes in ocular structure [35].

## Conclusions

VCD is the principal component of axial elongation in pediatric myopia, with minor effects from AD and LT. Longitudinal monitoring of VCD, combined with AL, may enhance individualized myopia management strategies, though further validation in larger cohorts is warranted. Looking forward, our findings on the strong association between VCD and axial elongation suggest that these parameters could be valuable components in future models aimed at predicting myopia progression.

## Data Availability

The datasets used and analyzed during the current study are available from the corresponding author on reasonable request.

## Abbreviations

AL: Axial length
CCT: Central corneal thickness
AD: Aqueous chamber depth
LT: Lens thickness
VCD: Vitreous chamber depth
SE: Spherical equivalent
